# XPS–SEM/EDS Tandem Analysis for the Elemental Composition of Functionalized Graphene Nanoplatelets

**DOI:** 10.1021/acsomega.5c07830

**Published:** 2025-11-07

**Authors:** Giovanni Chemello, Jörg Radnik, Vasile-Dan Hodoroaba

**Affiliations:** 42220 Federal Institute for Materials Research and Testing (BAM), Unter den Eichen 44-46, 12203 Berlin, Germany

## Abstract

Over the past decade, energy-dispersive X-ray spectrometry (EDS) with scanning electron microscopy (SEM) has advanced to enable the accurate analysis of light elements such as C, N, or O. For this reason, EDS is becoming increasingly interesting as an analytical method for the elemental analysis of functionalized graphene and could be an attractive alternative to X-ray photoelectron spectroscopy (XPS), which is considered the most important method for elemental analysis. In this study, comparative XPS and EDS investigations under different excitation conditions are carried out on commercially available powders containing graphene particles with different morphologies. The slightly different XPS/HAXPES and EDS results can be explained by the different information depths of the methods and the functionalization of the particle surfaces. For the material with smaller graphene particles and higher O/C ratios, all methods reported a lower O/C ratio in pellets compared with the unpressed powder samples. This clearly shows that sample preparation has a significant influence on the quantification results, especially for such a type of morphology. Overall, the study demonstrates that EDS is a reliable and fast alternative to XPS for the elemental quantification of functionalized graphene particles, provided that differences in the information depth are taken into account. Particle morphology can be examined in parallel with quantitative element analysis, since EDS spectrometers are typically coupled with SEM, which are available in a huge number of analytical laboratories.

## Graphical abstract

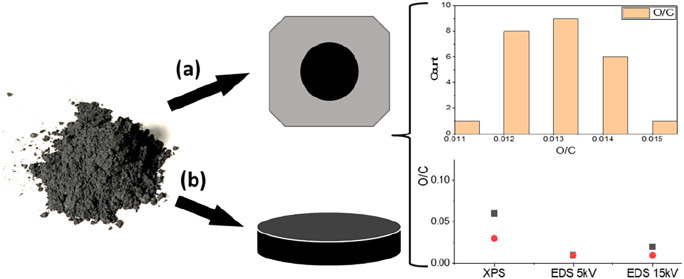

## Introduction

From its first isolation in 2004,^1^ graphene attracted the interest of the scientific community due to its numerous outstanding properties,^2^ such as unique electronic properties,^3^ extreme mechanical strength,^4^ exceptionally high thermal conductivity,^5^ impermeability to fluids,^6^ possibility of functionalization,^7^ and many other supreme properties, which makes graphene and its derivatives as suitable materials for various potential applications.^2^ Examples of attractive applications range from biosensors, therapeutics, and tissue engineering in the biomedical field,^8^ graphene-based transistors and integrated circuits in the electronic field,^9^ integration in photovoltaic modules, fuel cells, batteries, and supercapacitors for energy conversion and storage,^10^ and filters for water purification and desalination.^11^

In recent years, graphene has transitioned from a laboratory-confined scientific setting to commercial mass production at an industrial scale. Numerous manufacturing companies are striving to establish themselves in the emerging graphene market to commercialize graphene-related two-dimensional (2D) materials (GR2M) or products and technology based on GR2M. However, such a technology transition needs time, and many challenges have not yet been overcome.^12^

GR2M can be synthesized from different raw materials^13^ and by several different processes,^14–16^ which are generally subdivided into two main approaches: top-down and bottom-up. Hence, the quality of the graphene products differs extremely, as it is highly dependent on the material sources, manufacturing techniques, and fabrication condition monitoring.^17^

This variability represents one of the key obstacles hindering the widespread adoption of graphene technology, i.e., the inconsistency of the quality of the industrially produced GRM, and especially, the low homogeneity and repeatability achieved between different batches.^17,18^

Studies such as^19,20^ highlight that a major fraction of the commercially available graphene has very poor quality and great variability of size, number of layers, defects, and purity, even containing a significant amount of graphitic nanoplatelets.

Different batch/material quality means different morphologies and chemistries and ultimately different properties; therefore, at this stage, it is practically impossible to develop technologies that take advantage of a specific property of pristine graphene in a scalable and cost-effective way.

To overcome this limitation and to move forward with the technological transition of industrial graphene, it is crucial to develop reliable, reproducible, and shared measurement procedures to properly characterize and label GR2M relative to their physicochemical properties.^21^ One established method for the quantitative bulk analysis of graphene-related materials that is relatively easily applicable at the commercial material producer is the CHN/O elemental analysis. However, this method is unable to sense heterogeneity on the micro/nanometer scale (only batch-to-batch). This may be essential for many applications, such as in polymer composites, where the polymer–graphene interactions highly depend on the degree of functionalization of the individual sheets. Analytical methods able to probe (sub)­micro- and nanosized dimensions of material are asked.

X-ray photoelectron spectroscopy (XPS) is a commonly used technique for surface-sensitive chemical characterization of materials, including GR2Ms such as graphene, reduced graphene oxide, graphene oxide, and functionalized GR2M.^22–24^ XPS has an information depth of around 10 nm, which is a similar dimension scale as the thickness of particles and platelets of GR2M consisting of a few monolayers. Recently, photoelectron spectroscopy studies combining soft and hard X-rays (HAXPES) indicate the potential to increase the probing depth up to about 20 nm.^25^

However, XPS/HAXPES analysis can be long and tedious due to the nanopowder nature of the samples, which might create issues with the ultrahigh vacuum working condition of the instrument.

Energy-dispersive X-ray spectroscopy (EDS) is a fast, easy-to-operate, and very widespread technique, which can be employed to run elemental analysis on different areas and, in combination with electron excitation at an SEM, combines chemical and morphological characterization. The recent study of Madbouly et al.^26^ also investigated the potential of EDS analysis in correlation with XPS along the production chain of commercial GR2Ms.

Pellets may substitute powder for the analysis of the chemical composition of GR2Ms, as they are easier to handle and safer for the operator, by reducing the risk of exposure to flying nanopowder, which may be hazardous for human health.^27^ Reed et al. carried out a study proposing and validating pelletization as a sample preparation procedure.^28^

In this study, we analyze by XPS/HAXPES and SEM/EDS two different graphene powders industrially synthesized through different approaches and both functionalized with oxygen by the same manufacturer using the same treatment. Both the raw materials and the O-functionalized samples were measured in the form of powder and pellets, see Figure 1. The results for all samples are then compared and correlated with the morphological information provided by SEM inspection. The latter highlights the different morphologies of the constituent particles of the two series of materials, raw and O-functionalized. The relationship between these different morphologies and chemical functionalization is discussed.

**Figure 1:**
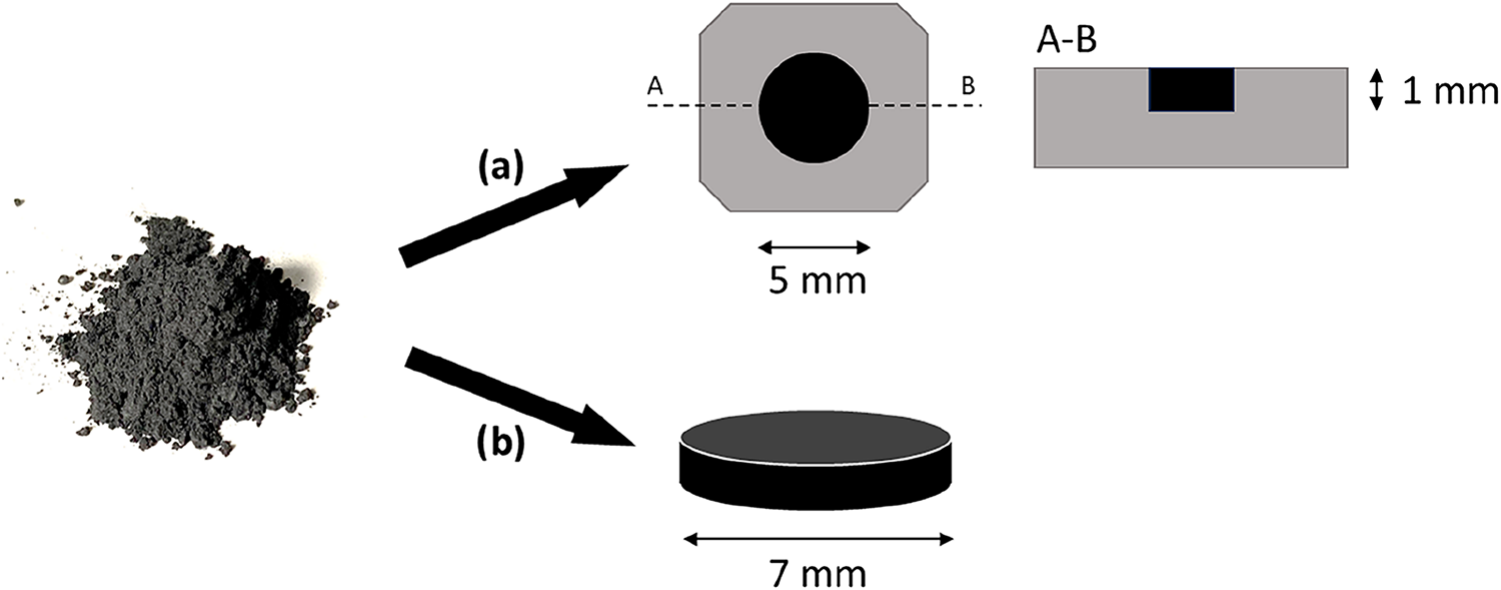
Example of a powder GR2M, as powder filling a recess (top) and a pelletized sample (bottom).

## Materials and Methods

### Materials

The materials used in this study are industrially produced plasma-functionalized graphene nanoparticles provided as powders by Haydale Ltd. (Ammanford, U.K.)^29^ and consist of two series of graphene particles and platelets of different morphologies labeled G5 and G6. The G5 material is characterized by larger, 2D-like particles and was produced using a top-down process, while G6 is characterized by smaller, rather three-dimensional (3D)-like particles and was produced using a bottom-up process. Each series comprises the corresponding raw unfunctionalized graphene powder (G5R and G6R) and the oxygen-functionalized graphene powder (G5O and G6O), see later the results and corresponding findings with Figure 3.

The graphene particles were functionalized by Haydale Ltd. using a plasma treatment.^30^ In short, commercially available few-layer graphene was introduced into a patented reactor barrel and placed into a HDPlas plasma reactor. Feed gas was introduced into a low-pressure chamber, where it was energized and ionized to create a plasma. The pressure and flow of the gas were regulated by a mass flow controller and a metered vacuum source. The reactor barrel both acted as a counterelectrode and rotated around the central electrode to facilitate mixing. The reaction process includes pretreatment and post-treatment to ensure homogeneity of the starting material and final product.

### Sample Preparation

For both materials, a powder and a pelletized sample were analyzed. The powder samples were produced by filling the powder in a sample holder with a 5 mm-diameter recess of 1 mm depth, leveling the surface with a spatula, and gently pressing to obtain a regular and evenly flat sample surface parallel to the sample holder surface. In this way, the outer portion of the material has been removed to avoid possible alteration to the powder structure. The pellet samples were produced by pressing a small amount of powder in a compact, manually operated hydraulic hand-held press (Specac Ltd., Orpington, U.K.). After loading the round 7.0 (±0.1) mm-diameter pellet die with the powder, an indicated load of 2.00 (±0.05) tons was applied to produce a solid pellet.^28^ Before the measurement, all of the samples were kept in a desiccator under vacuum at a pressure of 100 Pa for 2 days to reduce the time required for the ultrahigh vacuum (UHV) pumping inside the instrument chamber.

### XPS and HAXPES Measurements

XPS and HAXPES measurements were performed using a lab-based ULVAC-PHI “Quantes” spectrometer (Chanhassen, MN, U.S.A.) equipped with two X-ray sources: a monochromatic Al Kα-source at 1486.6 eV for XPS and a monochromatic Cr Kα-source at 5414.8 eV for HAXPES. With this instrument, it was possible to perform XPS and HAXPES measurements at the exact same location on the sample. The X-ray beam spot size was set to 100 μm, and the photoelectrons were collected at an emission angle of 45° for both XPS and HAXPES. The Al Kα-source was situated normal to the sample, and the incident angle of the Cr Kα-source was 22°. The pressure in the sample chamber was kept below 10^–6^ Pa during the measurements. The measurements were performed in three different locations for each sample. Low-energy electrons and Ar^+^ ions were used for charge neutralization. The spectra were referenced to the C 1s binding energy of 284.5 eV. The XPS and HAXPES spectra were collected as survey spectra with a step size of 1 eV, a pass energy of 280 eV, and a time per step of 200 ms. The measurements were repeated with 2 sweeps for XPS at an X-ray power of 25 W at 15 kV. Here, the binding energy range selected was from 0 to 1100 eV for the survey spectra. For the HAXPES survey spectra, the repetition rate was set to 10 sweeps at an X-ray power of 50 W at 20 kV. The binding energy range was set from 0 to 2000 eV. The measurements were performed at three different points on each sample. For the quantification of the atomic concentration, PHI MultiPak Software Version 9.9.1 was used, with relative empirical sensitivity factors provided by the manufacturer. The binding energy scale was calibrated following a PHI procedure, which uses the ISO 15472 binding energy data.^31^ The intensity was calibrated with the PHI MultiPaK software following a procedure introduced by Seah.^32^ A Shirley background was used.

### SEM/EDS Measurements

The measurements were carried out using an EDS spectrometer from Oxford Instruments with an EDS SDD (silicon-drift detector) detector of 30 mm^2^ crystal area, which is attached to an SEM of type Hitachi FlexSEM. The quantification was carried out with the Aztec software (Oxford Instruments) version 5.0 (2020) in the standardless version. To check the performance of the standardless quantification, the ionic liquid (IL) 1-propyl-3-methyl-imidazolium bis­(trifluoromethylsulfonyl)­imide (Solarpur, Germany) of well-known elemental composition was measured and quantified under the same conditions as the GR2Ms.^34–36^ XPS performs excellent with relative deviations of max 5% for all 5 elements. It is well-known that the (standardless) quantification of SEM/EDS for light elements is basically more inaccurate than that for elements above 11 (Na). This is so partly due to the higher uncertainties of the fundamental parameters associated with the characteristic X-ray lines of the light elements necessary for the quantification but partly due to the higher sensitivity of these low-energy X-ray lines to the sample surface morphology (e.g., powder vs pellet preparation) and inhomogeneities. Nevertheless, the technological developments in the past decade regarding the quality of SDD (silicon-drift detector) EDS systems (i.e., spectrometer efficiency and energy resolution), also including their thin film windows, have significantly contributed to this improvement. Thus, the results of quantification of the IL selected as a reference material with ideal homogeneity and surface planarity demonstrate the very good quality of the EDS quantification of C, N, O, F, and S of maximum 10% relative deviation, at both excitations of 5 and 15 kV.^37^

The spatial information corresponding to the SEM/EDS analyses on the selected IL with the chemical composition as above varies from ∼1.5 μm at 15 kV down to ∼200 nm at 5 kV; see the representation in Figure 2 resulting from Monte Carlo simulations.

**Figure 2:**
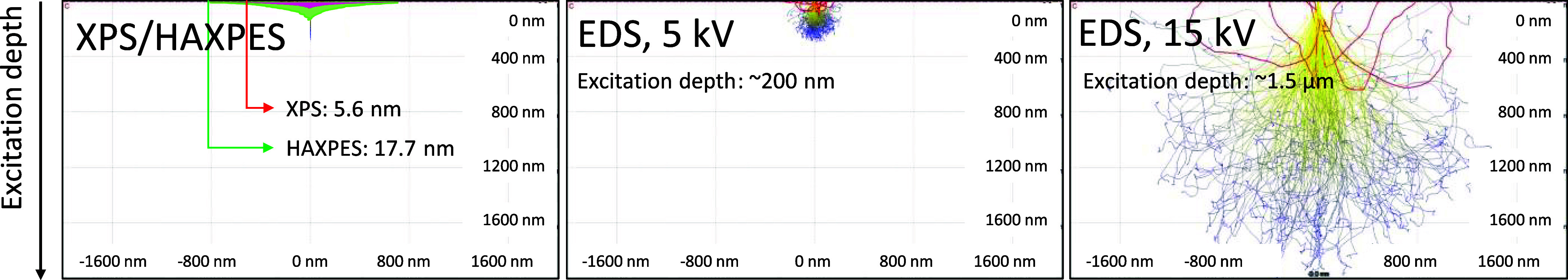
Schematic visualization of the depth resolution attained in a 100%-carbon material with XPS, HAXPES, SEM/EDS at 5 kV, and SEM/EDS at 15 kV. The SEM/EDS results are obtained through Monte Carlo simulations for electrons (CASINO, version 2.42).

The samples were measured at three locations of around 150 μm × 150 μm area each.

### SEM Measurements

The morphology analysis of the powders was carried out using an SEM instrument of type Zeiss Supra 40 (Oberkochen, Germany), with a Schottky field emitter and a secondary electron *InLens* detector, to obtain high-resolution images of the powder samples’ surface.

## Results and Discussion

Figure 3 shows an overview of SEM images for each sample in both forms as powder and pellet. By comparing the two GR2M series, G5 and G6, at two different magnifications, it appears clear that the two materials present a remarkably different morphology.^38^ G5 presents bigger (mostly >10–100 μm), jagged, and platelet-looking particles, while G6 has smaller (∼10 μm), regular, and almost round-shaped particles. This difference in morphology remains unchanged for both the unfunctionalized and the O-functionalized samples.

**Figure 3:**
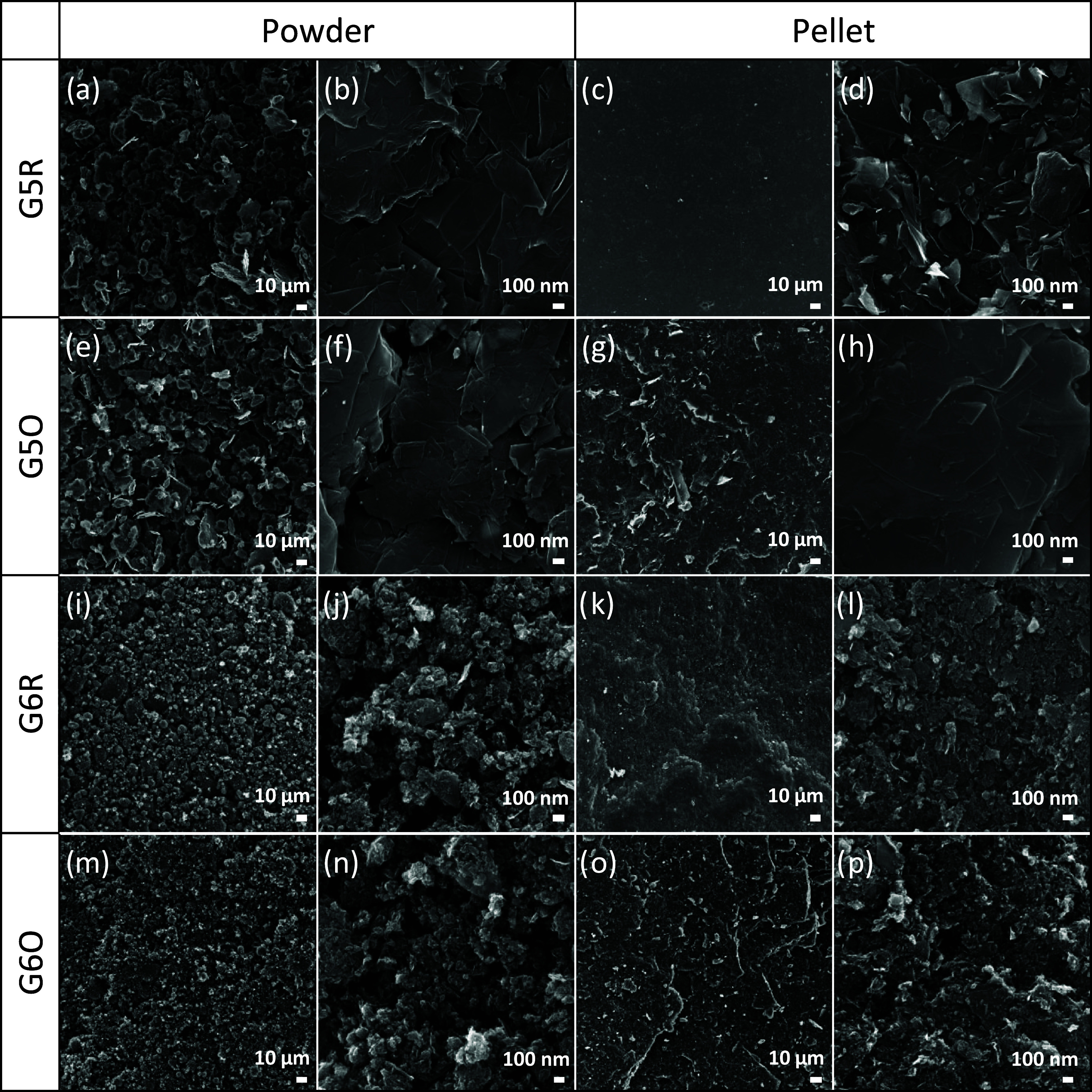
Overview of SEM images comparing the morphology of all of the samples at two different magnifications. (a, b) G5R in powder form, (c, d) G5R as pellet, (e, f) G5O as powder, (g, h) G5O as pellet, (i, j) G6R as powder, (k, l) G6R as pellet, (m, n) G6O as powder, and (o, p) G6O as pellet.

The morphology of the pellet samples, in comparison with the powder ones, is characterized by an overall lower porosity and roughness. At low magnification, the pelletized samples present a rather homogeneous and regular surface morphology, and at high magnification, the particles still show the same shape features of the respective powder material (G5 or G6), while looking significantly more tightly packed.

In Table 1 and Figure 4, the mean atom-% O values for each sample, measured by XPS, HAXPES, EDS 5 kV, and EDS 15 kV, are represented.

**Figure 4:**
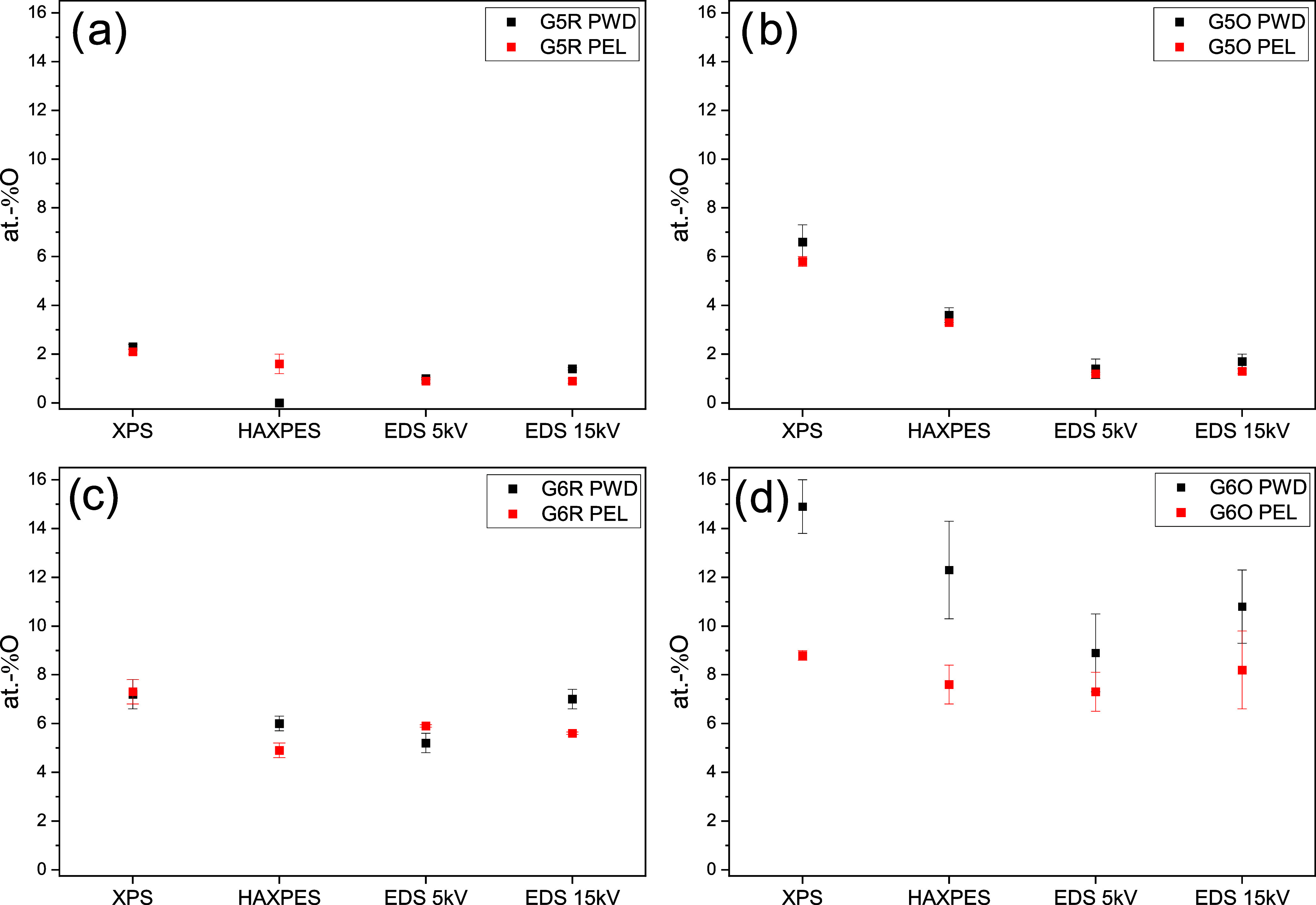
Comparison of the mean atom-% O for each material (G5 and G6), functionalization type (raw material and O-functionalization), and preparation route (powder, PWD, and pellet, PEL), resulting from XPS, HAXPES, EDS 5 kV, and EDS 15 kV analyses. Specifically, (a) G5R powder and pellet samples, (b) G5O powder and pellet, (c) G6R powder and pellet, and (d) G6O powder and pellet.

**Table 1:** Summary of the Calculated Atom % O Mean Value for Each Material (G5 and G6), Functionalization Type (Raw Material and O-Functionalization), and Preparation Route (Powder and Pellet) Resulting from XPS, HAXPES, EDS 5 kV, and EDS 15 kV Analyses^a^

		O atomic fraction (atom-%)			
		G5R	G5O	G6R	G6O
XPS	powder	2.3 (0.1)	6.6 (0.7)	7.2 (0.6)	14.9 (1.1)
	pellet	2.1 (0.3)	5.8 (0.2)	7.3 (0.5)	8.8 (0.2)
HAXPES	powder	traces	3.6 (0.3)	6.0 (0.3)	12.3 (2.0)
	pellet	1.6 (0.4)	3.3 (0.1)	4.9 (0.3)	7.6 (0.8)
EDS 5 kV	powder	1.0 (0.0)	1.4 (0.4)	5.2 (0.4)	8.9 (1.5)
	pellet	0.9 (0.1)	1.2 (0.0)	5.9 (0.0)	7.3 (0.8)
EDS 15 kV	powder	1.4 (0.0)	1.7 (0.3)	7.0 (0.4)	10.8 (1.5)
	pellet	0.9 (0.1)	1.3 (0.1)	5.6 (0.0)	8.2 (1.6)

a In brackets, the standard deviation is also given.

The atom-% O found by all methods on the unfunctionalized samples G5R in Figure 4a shows comparable values in the range of 1–2 atom-% O for both the powder and pellet samples. The highest O content was detected by XPS on both powder and pellet samples, respectively, 2.3 and 2.1 atom-%. The lowest was detected by HAXPES on the powder sample, where a value lower than the instrument sensitivity (<1 atom-%) was found.

Differently from the unfunctionalized sample of the same series, in the case of the O-functionalized sample G5O in Figure 4b, some relevant differences were found in the atom-% O values detected by the instruments. While the atom-% O values found for both powders and pellets by EDS 5 and 15 kV are again comparable to each other and rather comparable to the G5R values, the atom-% O found by XPS and HAXPES for the powder and pellet samples are higher. In particular, the values measured by XPS on both powder and pellet samples result in atom-% O around three times higher than the EDS ones and almost double the values measured by HAXPES. The higher atom-% O content compared to G5R is not surprising since G5O is, in fact, an O-functionalized material; however, this difference is highlighted only by XPS and HAXPES. Considering the extremely low information volume of the XPS method in comparison to SEM/EDS, this may suggest that the functionalization takes place mainly at the first 10 nm of the surface.

Moving on to the G6 series, and specifically to the unfunctionalized sample G6R in Figure 4c, the atom-% O measured by all methods shows again comparable values, similar to the trend of the G5R sample. However, in case of the G6 series, the O mean values range between 5 and 7 atom-%, revealing that the G6 is an O-richer raw material compared to G5. This increased presence of oxygen in G6 can be explained by its higher specific surface area,^28^ also highlighted by the SEM micrographs in Figure 3.

The O-functionalized sample G6O in Figure 4d presents the highest recorded O atom-% among all of the sample series, with the highest value measured by XPS, followed by HAXPES, and then comparable values for both EDS measurements. The same trend applies for both powder and pellet samples; however, the former shows a higher O atom-% mean than the latter. In particular, the O atom-% means measured by XPS and HAXPES on the powder samples are almost the double that of the pellet ones, while this difference for the O atom-% measured by EDS 5 and 15 kV is considerably lower and almost comparable. The significantly higher O atom-% mean value found by XPS on the powder sample in comparison with the pellet one may suggest that the pelletization process reduces the surface-located functionalization.

Additionally, small traces (<1 atom-%) of other elements, such as iron, silicon, and aluminum, were found by the EDS analyses, which are not reported in the graph or in the table.

The fair agreement of the O/C values of all of the pellet samples calculated by EDS at 5 and 15 kV and their relatively small uncertainty indicate that the pellets are homogeneous. In a further analysis, Figure 5 shows an example of the SEM/EDS 5 × 5 grid analysis performed on pellet samples: G5R at 5 kV. The calculated atom-% C and atom-% O shown for each location show homogeneous values and a resulting atom-% O SD of 0.1 (RSD ≈ 7%). Equivalent results were found for all of the other pellet samples with a RSD of max ≈10% for each 5 × 5 grid analysis.

**Figure 5:**
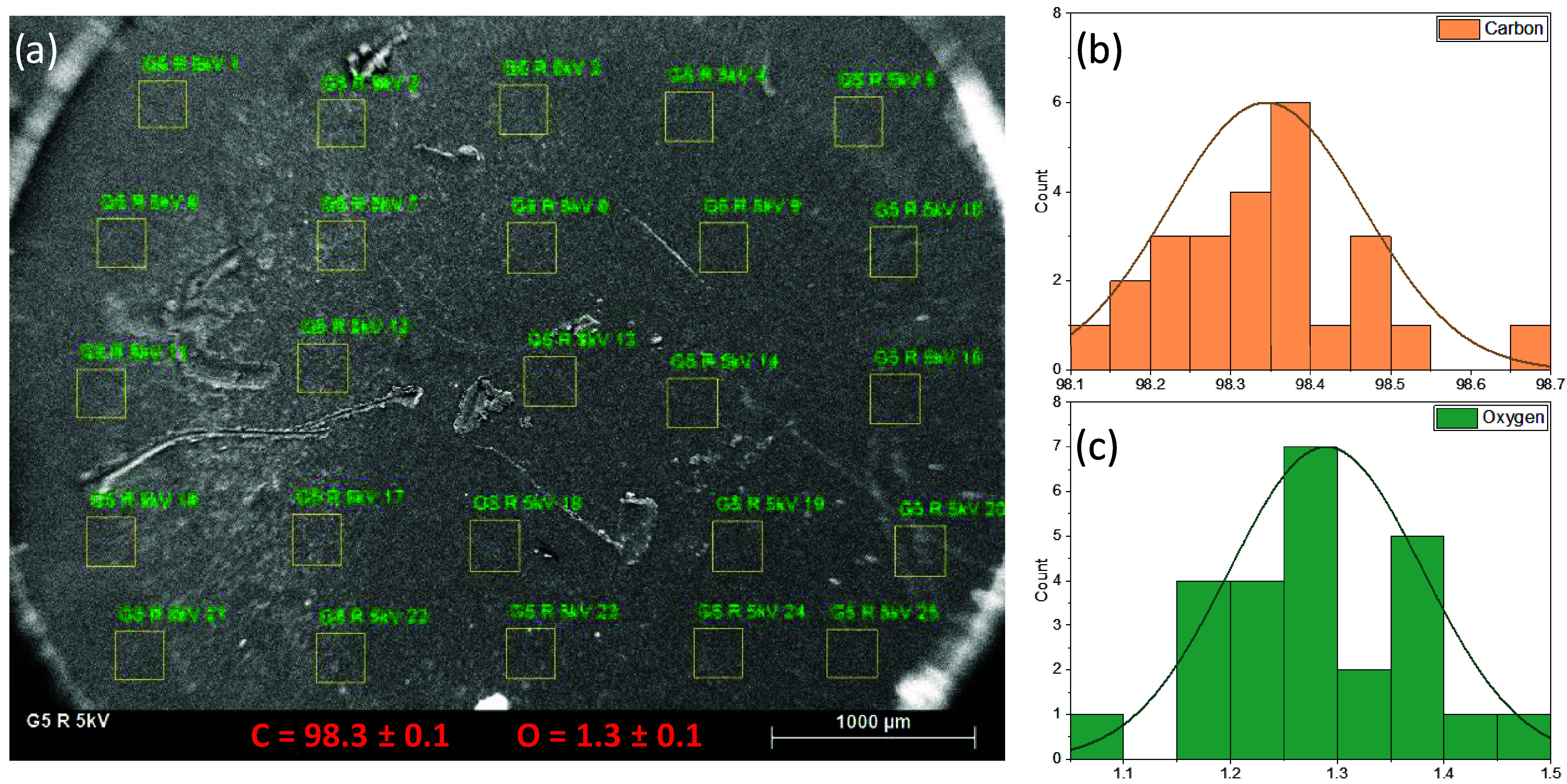
Example of a 5 × 5 grid analysis performed by SEM/EDS on all of the pellet samples. (a) Pellet sample G5R analyzed at 5 kV showing all of the locations of the grid analysis and the resulting mean C atom-% and O atom-% in red at the bottom. (b, c) Histograms of the O and C atom-% resulting from the 5 × 5 grid analysis.

As presented in this study, commercially available graphene-related materials show noticeable variation in morphological structure, elemental composition, and degree of functionalization depending on their processing histories. It is important to note that all of the powders analyzed in this work were provided by a single supplier. Therefore, the results may not be representative of materials from different origins, which exhibit varying impurity profiles and degrees of homogeneity.

## Conclusions

In this paper, two types of graphene materials of different morphologies were investigated, i.e., the first with a more 2D-like structure and larger (μm) size and the second with a rather nanoparticulate shape. The oxygen-functionalized version of the two materials was compared with the unfunctionalized samples.

In the case of the unfunctionalized samples, no major differences were observed in the oxygen content as detected by XPS, HAXPES, EDS at 5 kV excitation, and EDS at 15 kV excitation, where all techniques have resulted in comparable O atom-% values.

For the functionalized samples, clearer differences were observed. The sample with larger nanoplatelets seems to be functionalized at the outermost surfaces of the stacks of the nanoplatelets, which can be seen in the decreasing O/C ratios from XPS via HAXPES to EDS. Slight differences were observed between the powders and pellets for XPS.

In the case of the oxygen-functionalized samples with smaller particles, the highest O/C ratios were observed. Whereas with XPS and HAXPES, a higher oxygen amount (almost double) was detected for the powder than for the pellets, the oxygen content detected by EDS 5 and 15 kV shows again comparable results for both powder and pellet. Such a dependence on the information depth of the methods cannot be observed for the pellets.

Pelletizing the powders led to smaller O/C ratios, especially for the sample with smaller graphene particles and for the most surface-sensitive technique, XPS. This should be considered in the analysis of the results. Therefore, the sample preparation and the morphology of the graphene particles next to the measurement conditions and the technique should always be reported with the measured O/C ratios.
